# Assessment of thickened endometrium in tamoxifen therapy

**DOI:** 10.4274/tjod.82621

**Published:** 2014-12-15

**Authors:** Engin Korkmazer, Neşe Solak, Vehbi Yavuz Tokgöz

**Affiliations:** 1 Giresun University Faculty of Medicine, Department of Gynecology and Obstetrics, Giresun, Turkey; 2 Giresun Maternity and Chilt Health Hospital, Clinic of Gynecology and Obstetrics, Giresun, Turkey; 3 Kızıltepe State Hospital, Clinic of Gynecology and Obstetrics, Mardin, Turkey

**Keywords:** Tamoxifen, hysteroscopy, dilatation and curettage, polyp

## Abstract

**Objective:**

Aim of this study was to evaluate role of hysteroscopy in thickened endometrium (>5 mm) associated with tamoxifen therapy.

**Materials and Methods:**

We performed dilatation and curettage (D&C) and hysteroscopic biopsy to patients for evaluation of thickened endometrium in tamoxifen therapy. One hundred and nine asymptomatic patients with estrogen receptor positive breast cancer treated with tamoxifen 20 mg daily. We performed hysteroscopic biopsy or D&C to patients who have thickened endometrium at transvaginal sonography. We correlate pathology report results of D&C and hysteroscopic biopsy.

**Results:**

Fifty-nine of 103 patients have thickened endometrium.Thirty-five of 59 patients diagnosed with D&C (19 inactive endometrium, 15 endometrial polyp, 1 endometrial hyperplasia). D&C couldn’t get material 24 of these patients. Hysteroscopic biopsy diagnosed endometrial polyp 11 (45.8%) of these patients.

**Conclusion:**

We can state that D&C does not seem accurate enough for detection of intrauterin pathologies in thickened endometrium associated with tamoxifen therapy. We therefore believe it is reasonable to perform hysteroscopic biopsy in asymptomatic tamoxifen treated patients who have thickened endometrium.

## INTRODUCTION

Tamoxifen is a nonsteroid antiestrogen, which has been used in chemoprevention of receptor-positive breast cancer^(1)^. It has antiestrogenic effects in breast tissue and estrogenic effects on endometrium and myometrium. Long-term administration of tamoxifen makes several changes in the endocervix and endometrium (atrophy, polyposis, proliferation, vascular changes^(2)^. The most serious effect on endometrium is increasing risk of developing endometrial carcinoma^(3,4)^. Therefore, even if they are asymptomatic, these patients must be evaluated carefully.

Transvaginal ultrasonography, sonohysterography, dilatation and curettage (D&C) and hysteroscopy have been used in the examination of tamoxifen-treated women with increased endometrial thickness (≥ 5 mm). Controversy exists regarding the most optimal method of screening. The value of Tv US in assesing endometrial thickness and endometrial charecteristics improved with technological developments, however it hasn’t yet achieved sufficient sensitivity and specificity in patients with abnormal endometrial thickness^(5,6)^. Saline infusion sonohysterography is another method that has been proposed for the evaluation of such patients, but didn’t reached desired value^(7)^. Although, D&C has beeen used as gold standard for many years, hysteroscopic evaluation and direct biopsy from the lesion has become superior to all aproaches^(8,9)^. In our study, asymptomatic patients with increased endomtrial thickness in whom pathological specimen couldn’t be obtained during D&C were further evaluated by hysteroscopy and we present these hysteroscopic findings.

## MATERIALS AND METHODS

The study population consisted of 103 asymptomatic women attending Zübeyde Hanım Maternity Hospiatal in Turkey. They were recieving 20 mg of tamoxifen daily for at least one year because of estrogen receptor positive breast cancer. Charecteristics like age, gravida, Body mass index (BMI), duration of tamoxifen therapy and endometrial thickness were noted. Informed written consent was taken from all patients and details of the study were explained.

After recording medical history and routine gynocological examinatoin, endometrial thickness was measured by TvUS (5.0-MHz Aloka 500 system). The uterus was scanned sagitally in order to get anteroposterior measurements of endometrial axis view between the outermost edges of the line separating the hyperechogenic endometrium from the myometrium. The maximal width was recorded and endometrial thickness cut off ≥5 mm was set as criterion for significant abnormality. Fifty four patients had thickness in normal range and further evaluation was needless. In 59 patients (57.2%) endometrium was ≥5 mm and they attempted D&C. Pathology specimens were fixed in 10% formaldehyde, paraffin blocks prepared and after Hematoxylin and Eosin stain was performed. All histological samples were analysed by a single specialist gynaecological pathologist to ensure consistency of interpretation within trial.

In endometrium thickenned group (n=59), pathology specimen was obtained in 35 (59.3%; specimen-positive group) patients during D&C and in 24 (40.7%) women specimen couldn’t be obtained (specimen-negative group).

Further evaluation and biopsy was performed in specimen-negative group by hysteroscopy (Karl-Storz 3 mm telescope with 300 oblique lens, Karl Storz GmbH, Tuttlingen, Germany). Vaginosopic hysteroscopy was performed and well tolerated by our patients and no anesthesia was used. The vagina and uterus was distended with 0.09% saline and endometrial cavity was visualized systematically beginning from tubal ostiums. From suspicious endometrial lesions biopsies were taken and all diagnosis were confirmed by biopsies. During D&C and hysteroscopy no compliactions were seen.

## RESULTS

One hundred and three tamoxifen recieving women for at least one year were enrolled in the study. Fifty nine of these womens had endometrial thickenning and the mean width was 11±5 mm and the mean age of women was 52± 8.3 (43-74) and mean BMI was 27±4.7 kg/m2. The demographic charecteristics are presented on Table 1.

In specimen-positive group (n=35), most frequent histopathological finding was inactive endomtrium (n=19, 54.2%) and second frequent diagnosis was endometrial polyp (n=15, 42.8%). Only one patient curettage revealed endometrial hyperplasia without atypia Table 2.

In specimen-negative group (n=24), that attempted hysteroscopy, biopsy findings were inactive endometrium (n=13, 55.2%) and endometrial polyp (n=11, 45.8%) (Table 2). The group’s mean endometrial thickness was 8±3 mm.

According to these results; in 40.6% of patients with endometrial thickenning, during D&C specimen wasn’t obtained and they needed further evaluation with hysteroscopy.

## DISCUSSION

As adjuvant therapy, Tamoxifen reduces mortality rates among estrogen reseptor positive breast cancer patients^(10)^. Due to estrogenic effects on endometrium, tamoxifen increases risk for endometrial pathologies^(3)^. Although, there is still controversary in follow up of asymptomatic tamoxifen recieving patients^(11)^.

In present study, endometrial cut off width was accepted as 5 mm, patients having values above ≥5 mm attempted invasive procedures (D&C, Hysteroscopic biopsy). In 27 (45.7%) of patients with endometrial thickenning (n=59), endomtrial pathologies were detected and it was consistent with literature^(12)^. But on the other hand, more than half of the patients (n=32, 54.3%) had invasive procedures although they didn’t have an underlying endometrial pathology.

In relation to proliferative effects of Tamoxifen on endoemtrium, Tamoxifen induces endometrial pathologies that can progress to endometrial cancer^(2)^. In present study, 1 endometrial hyperplasia and no cases of cancer were detected. However, in high risk populations for endometrial cancer, such as patients undergoing Tamoxifen theraphy, its recomended to use biopsy and hysteroscopic evaluation in combination^(13)^. Although its assumed that hysteroscopic examination in high risk patients for cancer, may increase the risk of dissemination of malignant cells into the peritoneal cavity, in recent meta-analyse no evidence was found to support an association between hysteroscopic examination and peritoneal cytology^(14)^.

Quareshi et al, supported the association between prolonged tamoxifen therapy and endometrial pathology^(15)^. In our study, there was no corelation between duration of therapy and endometrial pathology (p=0.584).

Dilatation and curettage was assumed as gold standart for assessment of endometrium, but in present study, during biopsies pathology specimen wasn’t obtained in 40.6% (n=24) of patients and D&C missed 18.6% (n=11) of endometrial pathologies. This highlights the difficulty of obtaining accurate histological data in D&C and this method alone would not provide sufficient information, especially in high-risk women. At this point, hysteroscopic evaluation and directed biopsy stands out in endometrial assessment among Tamoxifen users. Sensitivity and specificity of hysteroscopy is especially high for space occupying lesions of endometrium but its not good in some circumstances like hyperplasia^(16)^. Larger studies on this issue recommend hysteroscopic evaluation must be supported with biopsy^(9)^. Also in our study, there was underlying pathology in 45.8% of patients that hystopathologic specimen couldn’t be obtained during curettage, so it’s reasonable to support D&C with hysteroscopic evaluation and biopsy.

The main point against hysteroscopy as screening test in women receiving tamoxifen is difficulty in access and its limited acceptability from the patients. With development of modified approaches such as very thin atraumatic hysteroscopes (2.9 mm), the rate of unsuccessfull procedures is decreasing^(17)^. In our series, during vaginoscopic hysteroscopy, speculum is not used and the cervix is not clamped or dilated, general or even local anesthesia is not required. All of our hysteroscopic procedures were completed successfully and well tolareted by patients.

The main limitations of our study is small sample size and we couldn’t determine the real sensitivity and specifity of hysteroscopy, because it was only performed in specimen negative group.

In conclusion; endometrial thickness during tamoxifen therapy must be evaluated carefully because of increased endometrial cancer risk and in evaluation of this endometrium, with improvements in technology, it seems hysteroscopy and hysteroscopic biopsy will take place of conventional D&C.

## Figures and Tables

**Table 1:**
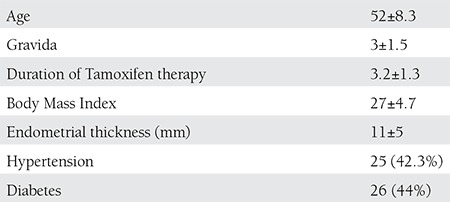
Demographic charecteristics of study group (n=59)

**Table 2:**
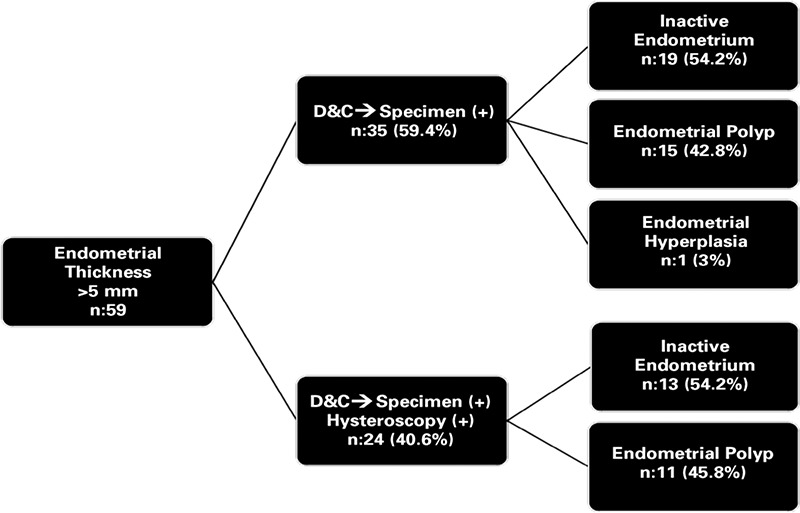
Distrubition of pathology results in patients with endometrial thickness ≥5 mm
